# Longitudinal Association Between Internet Gaming Disorder and School Refusal Among Adolescents Using a Random Intercept Cross-Lagged Panel Model: Three-Wave Prospective Cohort Study

**DOI:** 10.2196/89619

**Published:** 2026-03-04

**Authors:** Peng Zheng, Zi Tao, Luoxiang Fang, Tao Yao, Lingling Zhou, Yutian Wang, Yuannan Lin

**Affiliations:** 1 Second Affiliated Hospital & Yuying Children's Hospital of Wenzhou Medical University, No. 109 Xueyuan West Road, Wenzhou, Zhejiang, 325000, China, 86 18358730598

**Keywords:** adolescent gamers, internet gaming disorder, school refusal, reciprocal association, sex differences

## Abstract

**Background:**

Internet gaming disorder (IGD) and school refusal are increasingly prevalent during adolescence, yet limited research has examined how they influence each other over time. Moreover, it is unclear whether the association differs by sex.

**Objective:**

This study aimed to investigate the within-person longitudinal associations between school refusal and IGD and potential sex differences in these associations among Chinese adolescent gamers.

**Methods:**

A 3-wave prospective cohort longitudinal study was conducted among students from 4 middle schools in Zhejiang Province, China. Adolescents who had played online games in the past 12 months were involved. Participants were recruited using convenience sampling. Data were collected at 6-month intervals: time 1 (T1, March 2024), time 2 (T2, September 2024), and time 3 (T3, March 2025). A total of 918 Chinese adolescents (n=609, 66.3% male; mean 13.55, SD 0.75) participated in the 3-wave survey. IGD and school refusal were assessed using the 9-item DSM-5 (*Diagnostic and Statistical Manual of Mental Disorders, Fifth Edition*) IGD checklist and the School Refusal Assessment Scale-Revised. A random intercept cross-lagged panel model and multigroup analyses were conducted.

**Results:**

All statistical tests were 2-tailed with *α*=.05. The random intercept cross-lagged panel model demonstrated good fit to the data (*χ*²_5_=16.02; comparative fit index 0.970; root mean square error of approximation 0.035). Cross-lagged effects indicated a reciprocal association between school refusal and IGD. School refusal predicted later IGD (T1→T2: *β*=.06, 95% CI .02-.08; *P*=.02 and T2→T3: *β*=.06, 95% CI .02-.08; *P*=.02), and IGD predicted subsequent school refusal (T1→T2: *β*=.12, 95% CI .09-.15; *P*<.001 and T2→T3: *β*=.13, 95% CI .08-.16; *P*<.001). Multigroup analyses revealed significant sex differences. Wald tests showed sex-specific effects: among females, school refusal predicted later IGD (T1→T2: *β*=.10, 95% CI .05-.15; *P*=.006 and T2→T3: *β*=.11, 95% CI .04-.17; *P*=.004), whereas these paths were nonsignificant for males. IGD predicted later school refusal in both sexes, but the effect was stronger for males (T1→T2: *β*=.15, 95% CI .12-.18; *P*<.001 and T2→T3: *β*=.15, 95% CI .12-.18; *P*<.001) than females (T1→T2: *β*=.07, 95% CI .03-.10; *P*=.02 and T2→T3: *β*=.07, 95% CI .03-.10; *P*=.02).

**Conclusions:**

This study provides novel insights into the reciprocal within-person associations between school refusal and IGD among adolescents. By accounting for stable between-person differences, the model clarifies how within-person fluctuations in one behavior relate to subsequent changes in the other. Sex-specific patterns were observed: school refusal predicted subsequent IGD among females, whereas IGD predicted later school refusal more strongly among males. These findings highlight the dynamic interplay between school disengagement and problematic gaming and underscore the importance of sex-sensitive intervention strategies.

## Introduction

Internet gaming disorder (IGD) refers to persistent and maladaptive engagement in online gaming that leads to significant functional impairment and is increasingly recognized as a pressing public health concern [1]. IGD is characterized by impaired control over gaming, prioritization of gaming over other responsibilities, and sustained engagement despite negative consequences [2]. Prevalence estimates highlight its public health relevance during adolescence, with meta-analyses reporting pooled rates ranging from 6.7% to 8.8% [3,4], and studies across different regions document similar elevated levels [5]. In China, a recent meta-analysis reported an IGD prevalence of approximately 10% [6]. IGD has been associated with numerous psychological and social difficulties, including depressive symptoms, loneliness, aggressive behaviors, and strained family relationships [7-10]. These patterns highlight that IGD encompasses broader developmental risks beyond excessive gaming alone. Given its multifaceted impact, understanding the processes that may reinforce or exacerbate IGD is essential. One understudied yet potentially important factor is school refusal, which may interact dynamically with IGD and form reciprocal influences over time, particularly during adolescence.

School refusal refers to difficulties in attending or remaining in school, including tardiness, skipping classes, morning misbehaviors designed to avoid school, and attending school with significant distress or reluctance [11,12]. School refusal is often accompanied by marked negative emotions such as anxiety, fear, and emotional distress [13]. According to the Functional Behavioral Model [14], school refusal is maintained by 4 key reinforcement-based functions: avoidance of negative affectivity (ANA), escape from aversive situations (EAS), attention-seeking (AS), and PTR. Existing evidence suggests that school refusal is highly prevalent, with estimates ranging from 28% to 35% among children and adolescents in Western and global samples [15] and from 22.5% to 30% in various regions of China [16]. Importantly, adolescents who exhibit school refusal are more likely than their peers to develop subsequent behavioral and adjustment difficulties [17,18].

School refusal is closely related to IGD among adolescents. However, the directionality between these 2 behaviors remains unclear. Given these uncertainties, it is necessary to consider how school refusal and IGD might relate to each other in both directions. First, school refusal may increase IGD. According to escape-avoidance theory, individuals tend to withdraw from environments that elicit stress or negative affect [19]. Adolescents who experience academic pressure, social-evaluative concerns, or negative emotional states at school often show higher levels of school refusal [20]. Online gaming, which offers immersion, controllability, and immediate relief from distress, may thus become an attractive alternative to aversive school experiences, thereby school refusal and increased gaming engagement within a shared pattern of avoidance-oriented coping [21]. In other words, when school elicits persistent distress, gaming may function as an emotionally soothing and controllable environment, reinforcing avoidance tendencies and increasing adolescents’ susceptibility to IGD.

Second, IGD may similarly increase school refusal. According to self-determination theory [22], individuals have 3 basic psychological needs: autonomy, competence, and belonging. Gaming environments frequently satisfy these needs through structured challenges, opportunities for volitional action, and social interaction [23,24]. When these experiences are perceived as more rewarding than those available in school, adolescents may experience lower motivation or diminished engagement in academic settings [25]. Sustained involvement in gaming may further weaken school-related motivation by displacing academic goals, reducing tolerance for effortful learning, and amplifying the contrast between gratifying digital environments and demanding school contexts. Under such conditions, stronger gaming involvement may be accompanied by increased tendencies toward school disengagement or avoidance. Together, these theoretical perspectives suggest that school refusal and IGD may mutually reinforce each other over time.

Empirical findings may provide support for this potential bidirectional association between school refusal and IGD. Consistent with theoretical expectations, studies across different cultural contexts show that adolescents who struggle with school engagement are more likely to rely on gaming or digital media. For example, digital game addiction was positively associated with school refusal among adolescents in Turkey [21], and excessive internet engagement was strongly related to both truancy and medically certified absences in a large Finnish national sample of more than 86,000 students [26]. Research also indicates that problematic internet use is a salient external correlate of school refusal; a latent class study among Chinese adolescents found that youth with school refusal behaviors frequently use gaming or online activities to escape academic and social pressures [27]. Additional evidence from Japan further shows that problematic internet use is highly prevalent among adolescents clinically identified with school refusal [28]. Conversely, research on school-related protective factors shows that stronger school identification predicts lower IGD risk [29]. This finding indirectly suggests that weakened school connectedness, which is often observed among youth with school refusal, may heighten vulnerability to gaming-related problems.

Reviews and meta-analytic work also highlight consistent associations between problematic internet use and school-related outcomes. A recent review synthesizing 10 studies concluded that problematic internet use represents a risk factor for school refusal [13], although no meta-analysis to date has examined school refusal and IGD specifically. Longitudinal findings further illustrate that digital behaviors are relevant to school functioning: problematic social media use and problematic smartphone use have been associated with declines in academic performance [30,31], problematic social network site use has predicted subsequent school absenteeism [32], and problematic gaming has been prospectively linked to increased truancy [33]. Despite these findings, no longitudinal studies have directly examined the potential bidirectional association between school refusal and IGD. This gap highlights the need for research that clarifies how school refusal and IGD may co-develop over time.

Emerging evidence suggests that the association between school refusal and IGD across developmental pathways may differ by sex. Epidemiological studies consistently show that male adolescents exhibit a higher prevalence of IGD than females [34,35]. Furthermore, male participants with IGD tend to be more sensitive to gaming-related rewards, whereas female participants show stronger affective dysregulation and mood-related symptoms [36]. These sex-specific patterns in gaming motivation and emotional processes may contribute to differential pathways linking school-related distress and problematic gaming behavior. At the same time, sex differences have also been documented in school attendance problems. For instance, female students generally report higher levels of school nonattendance compared with male students [37]. Evidence from a latent profile analysis further indicates that although most school refusal profiles show no significant sex differences, females are overrepresented in the mixed school refusal behavior profile, albeit with a small effect size [38]. This suggests that while sex disparities in school refusal are small, females may be more likely to exhibit complex or multifaceted forms of school refusal.

Although no study has explicitly tested whether sex moderates the association between school refusal and IGD, the existing evidence provides a plausible basis for sex-specific pathways. School-related distress may have a stronger impact on IGD among females due to heightened emotional vulnerability [39], whereas IGD may more strongly contribute to school-related difficulties among males, given their higher reward sensitivity and greater IGD prevalence [40]. However, it remains unclear whether these potential sex-specific patterns develop over time. This study, therefore, examines sex differences in the longitudinal association between school refusal and IGD, with the aim of informing sex-sensitive prevention strategies and advancing understanding of underlying developmental processes.

To advance the understanding of how school refusal and IGD develop over time, the present study used a 3-wave longitudinal design among Chinese adolescent gamers. A random intercept cross-lagged panel model (RI-CLPM) was applied to disentangle stable between-person differences from within-person fluctuations. Focusing on the within-person level, this study aimed to clarify the reciprocal relationship between school refusal and IGD. Based on previous empirical findings and theoretical perspectives, three hypotheses were proposed: (H1) Increases in school refusal would predict higher IGD at the next time point. (H2) Higher IGD would predict increases in school refusal over time. (H3) The reciprocal within-person association between school refusal and IGD would differ between male and female adolescent gamers. Findings are expected to provide deeper evidence on how short-term changes in school refusal and IGD develop and to inform sex-sensitive prevention strategies.

## Methods

### Study Design, Participants, and Setting

This study was designed as a 1-year prospective cohort study and conducted among students from 4 middle schools in Zhejiang Province, China. Data were collected at three 6-month intervals: time 1 (T1, March 2024), time 2 (T2, September 2024), and time 3 (T3, March 2025). The target population comprised adolescents who had played online games in the past 12 months. The participating schools were selected using convenience sampling. In collaboration with school administrators, all eligible students in the selected classes were invited to participate. Trained field workers administered the survey during regular classroom sessions. Prior to completing the questionnaire, the field workers briefed the students on the objectives and procedures of the study, assured them of the anonymity and confidentiality of their responses, and emphasized the voluntary nature of participation. Parents or legal guardians were informed about the study in advance.

To enable linkage of repeated responses across the 3 assessment waves, students provided their school identification numbers, which were subsequently encoded for matching purposes. These identifiers were permanently removed from the analytical dataset after linkage to ensure participant anonymity.

At baseline (T1), 983 adolescents who met the inclusion criteria completed the survey. By the final wave (T3), 918 participants remained in the cohort (n=609, 66.3% male; mean 13.55, SD 0.75). Among the 918 participants who completed all 3 waves, baseline demographic variables had the following missing values: sex (2/918, 0.2%), father’s educational level (8/918, 0.9%), mother’s educational level (5/918, 0.5%), self-reported academic performance (9/918, 1.0%), and single-parent family status (3/918, 0.3%). There were no missing data for perceived family financial situation or gaming time per month. A test for missing completely at random indicated that the missingness was completely random.

### Ethical Considerations

Prior to each wave of data collection, detailed information regarding the aims, procedures, potential risks, and confidentiality protections of the study was provided to both students and their parents or legal guardians. Written informed consent was obtained from parents or legal guardians, and written assent was obtained from the students. Participation was entirely voluntary, and it was clearly communicated that students had the right to decline or withdraw from the study at any time without any academic or administrative consequences. All questionnaires were self-administered and completed anonymously. An information sheet outlining participants’ rights and data protection measures was distributed before survey administration. No incentive was provided to the students. No identifiable images of participants were collected or used in this study. The study protocol and consent procedures were reviewed and approved by the Institutional Ethics Committee of the Second Affiliated Hospital & Yuying Children's Hospital of Wenzhou Medical University (approval 2024-K-239‐02).

### Studied Variables and Measurements

#### Background Factors

Background factors were collected, including age, sex, parental educational level, self-reported academic performance, perceived family financial situations, single-parent family status, and gaming time per month.

#### IGD

IGD was assessed using the 9-item *DSM-5* (*Diagnostic and Statistical Manual of Mental Disorders, Fifth Edition*) IGD checklist [1]. The checklist captures the presence of IGD symptoms over the past 12 months (yes/no response). The 9 criteria include preoccupation with gaming, withdrawal, tolerance, loss of control, giving priority to gaming over other activities, persistence despite negative consequences, deception regarding gaming time, escapism, and functional impairment due to gaming. It has been validated among adolescents and demonstrated satisfactory psychometric properties [41]. In this study, the Cronbach α of IGD was 0.78 at T1, 0.84 at T2, and 0.81 at T3.

#### School Refusal

The School Refusal Assessment Scale-Revised (SRAS-R) was used to assess school refusal [11]. It is a 24-item scale comprising 4 dimensions: ANA, EAS, AS, and PTR. Sample items include the following: “How often do you have bad feelings about going to school because you are afraid of something related to school (eg, tests, school bus, teacher, fire alarm)?,” “How often do you stay away from school because it is hard to speak with the other kids at school?,” “How often do you feel you would rather be with your parents than go to school?,” and “When you are not in school during the week (Monday to Friday), how often do you leave the house and do something fun?.” The items are rated on a 7-point Likert scales (1=never to 7=always). It has been validated among adolescents and demonstrated satisfactory psychometric properties [42]. In this study, the Cronbach α of school refusal was 0.88 at T1, 0.90 at T2, and 0.94 at T3.

### Data Analysis

Attrition analyses were conducted to compare participants who completed all 3 waves with those who discontinued participation at follow-up. Independent-sample *t* tests were used to examine sex differences in the 4 school refusal dimensions and IGD, and Pearson correlations were performed among the key study variables. These preliminary analyses were performed in IBM SPSS Statistics (version 28.0) using listwise deletion, as they were primarily descriptive and exploratory in nature and aimed to summarize observed data based on complete cases.

To evaluate the temporal stability of the measurement models, longitudinal measurement invariance tests were conducted for both school refusal and IGD. Criteria for acceptable fit followed established recommendations [43], including comparative fit index (CFI) and Tucker-Lewis Index (TLI) values ≥0.90 and root mean square error of approximation (RMSEA) and standardized root mean square residual (SRMR) values ≤0.08. Metric and scalar invariance were assessed by examining changes in model fit across nested models, with ΔCFI≤0.01 and ΔRMSEA≤0.015 indicating invariance. Results supported longitudinally consistent factor structures, confirming both metric and scalar invariance across waves.

The reciprocal associations between school refusal and IGD were examined using RI-CLPM. In these models, school refusal was specified as a latent construct indicated by the 4 functional dimensions of the SRAS-R: ANA, EAS, AS, and PTR. The 4 subscale scores were used as observed indicators of a higher-order latent school refusal factor at each wave, consistent with the functional behavioral model [14] of school refusal and allowing the shared variance across dimensions to be captured at the construct level. IGD was operationalized as a continuous symptom count score by summing the 9 DSM-5 IGD checklist items, which is a commonly adopted approach in IGD research [44-46]. Prior to model estimation, observed variables were grand-mean centered so that intercepts reflected overall mean levels across the 3 assessment waves. Model specification began with an unconstrained RI-CLPM in which all autoregressive and cross-lagged paths were freely estimated across time. To obtain a more parsimonious and stable model, cross-lagged paths were then constrained to be equal across adjacent time intervals. In a subsequent step, both autoregressive and cross-lagged paths were constrained to be time-invariant, and model fit was compared across nested models to identify the optimal specification. After identifying the optimal model, covariates were incorporated as predictors of the between-person random intercepts, including age, sex, self-reported academic performance, perceived family financial status, and single-parent family status. Gaming time per month was included as a time-varying covariate at the within-person level, such that gaming time at each wave predicted the within-person deviations of school refusal and IGD at the corresponding time point, thereby controlling for concurrent fluctuations in gaming behavior.

The multigroup RI-CLPM approach was adopted to test potential sex differences in the longitudinal association between school refusal and IGD. An unconstrained model allowing all structural paths to vary across groups was compared with a model in which the cross-lagged effects were constrained to equality. A significant *χ*^2^ difference indicated the presence of sex-specific effects.

All structural equation models, including the RI-CLPM and multigroup models, were estimated using Mplus version 8.3 (Muthén & Muthén). Full information maximum likelihood estimation was used to handle missing data in these models, given its advantages in longitudinal structural equation modeling for producing unbiased parameter estimates and maximizing data utilization under the missing at random assumption [47]. Statistical significance was set at 2-tailed *P*<.05 for all analyses. This study was reported in accordance with the American Psychological Association Reporting Standards for Studies Using Structural Equation Modeling [48].

## Results

### Attrition Analyses

As shown in Table 1, the 2 groups did not differ significantly with respect to sex, father’s and mother’s educational level, self-reported academic performance, perceived family financial status, single-parent status, gaming time per month, age, ANA, EAS, AS, PTR, and IGD.

**Table 1.** Attrition analyses comparing adolescent gamers who completed all 3 waves of the longitudinal study and those lost to follow-up^a^.

Variables	Follow-up (n=918)	Lost to follow-up (n=65)	*P* value
Categorical variables, n (%)			
Sex			.38
Male	609 (66.3)	45 (69.2)	
Female	307 (33.4)	20 (30.8)	
Missing data	2 (0.2)	—^b^	
Father’s educational level			.57
Middle school or below	630 (68.6)	45 (69.2)	
High school or equal level	208 (22.7)	17 (26.2)	
University or above	72 (7.8)	3 (4.6)	
Missing data	8 (0.9)	—	
Mother’s educational level			.62
Middle school or below	656 (71.5)	47 (72.3)	
High school or equal level	171 (18.6)	14 (21.5)	
University or above	86 (9.4)	4 (6.2)	
Missing data	5 (0.5)	—	
Self-reported academic performance			.60
Bottom 20%	131 (14.3)	7 (10.8)	
21st-40th percentile	188 (20.5)	16 (24.6)	
41st-60th percentile	226 (24.6)	18 (27.7)	
61st-80th percentile	213 (23.2)	11 (16.9)	
Top 20%	151 (16.4)	13 (20)	
Missing data	9 (1)	—	
Perceived family financial situation			.36
Below average	119 (13)	9 (13.8)	
Average	622 (67.8)	39 (60)	
Above average	177 (19.3)	17 (26.2)	
Single-parent family status			.38
Yes	71 (7.7)	2 (3.1)	
No	682 (74.3)	51 (78.5)	
Choose not to report	162 (17.6)	12 (18.5)	
Missing data	3 (0.3)	—	
Gaming time per month			.59
<4 h	406 (44.2)	28 (43.1)	
4-8 h	257 (28)	19 (29.2)	
8-12 h	105 (11.4)	4 (6.2)	
12-16 h	93 (10.1)	8 (12.3)	
>16 h	57 (6.2)	6 (9.2)	
Continuous variables, mean (SD)			
Age (years)	13.55 (0.75)	13.65 (0.76)	.65
ANA^c^	12.43 (5.99)	12.55 (6.57)	.87
EAS^d^	10.21 (3.72)	9.77 (3.5)	.35
AS^e^	11.71 (5.87)	10.52 (5.55)	.12
PTR^f^	14.97 (6.36)	14.85 (6.5)	.88
IGD^g^	1.51 (1.72)	1.88 (2.16)	.10

a ANA, EAS, AS, and PTR are 4 dimensions of school refusal.

b Not available.

c ANA: avoidance of negative affectivity.

d EAS: escape from aversive situations.

e AS: attention-seeking.

f PTR: pursuit of tangible rewards.

g IGD: internet gaming disorder.

### Sex Differences in Main Variables

Male participants showed significantly lower mean scores (SD) than female participants across the 4 dimensions of school refusal across the 3 waves, except EAS at T1. In contrast, male participants showed significantly higher mean scores (SDs) than female participants in IGD across the 3 waves (Table 2).

**Table 2.** Sex differences in school refusal dimensions and internet gaming disorder (IGD) across 3 waves among Chinese adolescent gamers^a^.

Dimensions	Boys, mean (SD)	Girls, mean (SD)	*t* test (*df*)	*P* value	Cohen *d*
ANA^b^ at T1	11.86 (5.71)	13.48 (6.27)	−3.92 (914)	<.001	−0.27
EAS^c^ at T1	10.08 (3.64)	10.49 (3.87)	−1.57 (914)	.12	−0.11
AS^d^ at T1	11.38 (5.80)	12.28 (5.83)	−2.21 (914)	.03	−0.15
PTR^e^ at T1	14.57 (6.38)	15.70 (6.16)	−2.54 (914)	.01	−0.18
ANA at T2	11.60 (6.16)	14.21 (6.92)	−5.81 (914)	<.001	−0.40
EAS at T2	9.69 (3.73)	10.54 (4.11)	−3.15 (914)	.002	−0.22
AS at T2	10.52 (5.63)	12.00 (5.94)	−3.68 (914)	<.001	−0.26
PTR at T2	14.49 (6.68)	16.53 (7.07)	−4.28 (914)	<.001	−0.30
ANA at T3	12.16 (7.16)	15.64 (8.28)	−6.60 (914)	<.001	−0.45
EAS at T3	10.09 (5.05)	11.30 (5.10)	−3.42 (914)	<.001	−0.24
AS at T3	10.82 (6.50)	13.23 (6.94)	−5.16 (914)	<.001	−0.36
PTR at T3	14.98 (7.76)	17.21 (7.83)	−4.08 (914)	<.001	−0.29
IGD at T1	1.71 (1.80)	1.10 (1.46)	5.14 (914)	<.001	0.37
IGD at T2	1.62 (1.95)	1.19 (1.45)	3.44 (914)	<.001	0.25
IGD at T3	1.91 (2.31)	1.33 (1.71)	3.95 (914)	<.001	0.29

a ANA, EAS, AS, and PTR are 4 dimensions of school refusal.

b ANA: avoidance of negative affectivity.

c EAS: escape from aversive social-evaluative situations.

d AS: attention-seeking.

e PTR: pursuit of tangible rewards.

### Measurement Invariance Tests

Table 3 presents the results of longitudinal measurement invariance testing for school refusal and IGD. The configural invariance models again demonstrated acceptable fit, with CFI and TLI exceeding 0.90 and RMSEA and SRMR below 0.08, indicating that the basic factor structures of both constructs were stable across the 3 waves. Metric invariance was supported, as changes in fit indices (ΔCFI ≤0.01; ΔRMSEA ≤0.015) fell within recommended thresholds, suggesting that the factor loadings remained consistent over time. Scalar invariance was also established, with minimal declines in model fit (ΔCFI ≤0.01; ΔRMSEA ≤0.015), indicating equivalence of item intercepts across measurement occasions.

**Table 3.** Longitudinal measurement invariance test for school refusal and internet gaming disorder (IGD).

Variables and model	CFI^a^	TLI^b^	RMSEA^c^	SRMR^d^	ΔCFI	ΔRMSEA	ΔSRMR
SR^e^							
Configural invariance	0.957	0.947	0.043	0.034	—^f^	—	—
Metric invariance	0.952	0.941	0.043	0.038	0.005	0.000	0.004
Scalar invariance	0.948	0.937	0.045	0.041	0.004	0.002	0.003
IGD							
Configural invariance	0.942	0.931	0.038	0.039	—	—	—
Metric invariance	0.937	0.929	0.038	0.042	0.005	0.000	0.003
Scalar invariance	0.935	0.921	0.041	0.044	0.002	0.003	0.002

a CFI: comparative fit index.

b TLI: Tucker-Lewis Index.

c RMSEA: root mean square error of approximation.

d SRMR: standardized root mean square residual.

e SR: school refusal.

f Not available.

Measurement invariance across sex was examined for school refusal and IGD. The configural invariance models showed acceptable fit, with CFI values above 0.90 and RMSEA and SRMR values below 0.08, indicating similar factor structures for males and females. Constraining factor loadings across groups resulted in minimal changes in model fit (ΔCFI ≤0.01; ΔRMSEA ≤0.015), supporting metric invariance. Scalar invariance was further supported (ΔCFI ≤0.01; ΔRMSEA ≤0.015), suggesting that item intercepts were comparable across sex. These results indicate that observed sex differences reflect substantive variation rather than measurement bias. Corresponding results are presented in Table S1 in Multimedia Appendix 1.

### Correlations

As shown in Table 4, the 4 dimensions (ANA, EAS, AS, and PTR) of school refusal were positively intercorrelated across the 3 waves, with *r* ranging from 0.22 to 0.69 (all *P*<.001). Each dimension of school refusal was also significantly and positively associated with IGD at all waves, with *r* ranging from 0.21 to 0.40 (all *P*<.001).

**Table 4.** Pearson correlation coefficients among school refusal dimensions and internet gaming disorder (IGD) measured at 3 time points^a^.

	1	2	3	4	5	6	7	8	9	10	11	12	13	14	15
1. ANA^b^ at T1	1														
2. EAS^c^ at T1	0.63^d^	1													
3. AS^e^ at T1	0.66^d^	0.42^d^	1												
4. PTR^f^ at T1	0.52^d^	0.42^d^	0.43^d^	1											
5. ANA at T2	0.56^d^	0.36^d^	0.44^d^	0.36^d^	1										
6. EAS at T2	0.38^d^	0.42^d^	0.26^d^	0.24^d^	0.66^d^	1									
7. AS at T2	0.46^d^	0.23^d^	0.58^d^	0.28^d^	0.69^d^	0.49^d^	1								
8. PTR at T2	0.33^d^	0.22^d^	0.27^d^	0.51^d^	0.56^d^	0.48^d^	0.47^d^	1							
9. ANA at T3	0.50^d^	0.28^d^	0.44^d^	0.36^d^	0.65^d^	0.33^d^	0.54^d^	0.41^d^	1						
10. EAS at T3	0.37^d^	0.32^d^	0.23^d^	0.29^d^	0.46^d^	0.44^d^	0.37^d^	0.29^d^	0.71^d^	1					
11. AS at T3	0.44^d^	0.23^d^	0.52^d^	0.28^d^	0.51^d^	0.30^d^	0.66^d^	0.34^d^	0.79^d^	0.64^d^	1				
12. PTR at T3	0.34^d^	0.23^d^	0.29^d^	0.48^d^	0.41^d^	0.27^d^	0.36^d^	0.55^d^	0.67^d^	0.58^d^	0.60^d^	1			
13. IGD at T1	0.44^d^	0.32^d^	0.30^d^	0.31^d^	0.24^d^	0.29^d^	0.28^d^	0.33^d^	0.24^d^	0.25^d^	0.19^d^	0.21^d^	1		
14. IGD at T2	0.34^d^	0.24^d^	0.30^d^	0.28^d^	0.39^d^	0.31^d^	0.31^d^	0.23^d^	0.31^d^	0.28^d^	0.25^d^	0.24^d^	0.53^d^	1	
15. IGD at T3	0.31^d^	0.25^d^	0.29^d^	0.21^d^	0.36^d^	0.31^d^	0.30^d^	0.24^d^	0.40^d^	0.36^d^	0.33^d^	0.30^d^	0.48^d^	0.55^d^	1

a ANA, EAS, AS, and PTR are 4 dimensions of school refusal.

b ANA: avoidance of negative affectivity.

c EAS: escape from aversive social-evaluative situations.

d *P*<.001.

e AS: attention-seeking.

f PTR: pursuit of tangible rewards.

### RI-CLPM

**Figure 1.**
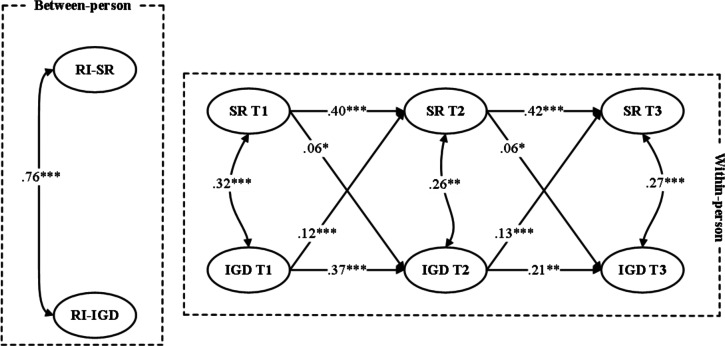
Random intercept (RI) cross-lagged panel model illustrating the longitudinal reciprocal associations between school refusal (SR) and internet gaming disorder (IGD) across 3 waves in a sample of Chinese adolescent gamers. School refusal was modeled as a latent construct indicated by 4 functional dimensions. The model separates stable between-person differences from within-person fluctuations over time. Standardized path coefficients are shown. β indicates standardized path coefficients. *P<.05, **P<.01, ***P<.001.

The fit indices of the final selected model are presented in Table S2 in Multimedia Appendix 1, demonstrating a good model fit to the data (*χ*^2^_5_=16.02; CFI=0.970; TLI=0.953; RMSEA=0.035; SRMR=0.029). The detailed results of model estimation are shown in Figure 1.

Within-person autoregressive paths were significant for both school refusal (T1 → T2: *β*=.40, 95% CI .29-.51; *P*<.001 and T2 → T3: *β*=.42, 95% CI .30-.54; *P*<.001) and IGD (T1 → T2: *β*=.37, 95% CI .16-.58; *P*<.001 and T2 → T3: *β*=.26, 95% CI .14-.38; *P*=.003), suggesting temporal stability.

Regarding cross-lagged effects, school refusal significantly predicted later IGD (T1 → T2: *β*=.06, 95% CI .02-.08; *P*=.02 and T2 → T3: *β*=.06, 95% CI .02-.08; *P*=.02). A reciprocal relationship was also observed, as IGD significantly and positively predicted school refusal (T1 → T2: *β*=.12, 95% CI .09-.15; *P*<.001 and T2 → T3: *β*=.13, 95% CI .08-.16; *P*<.001).

Within-person residual correlations among school refusal and IGD were positive and significant across all time points, with *r* ranging from .26 to .32. At the between-person level, the latent trait factors of school refusal and IGD were positively correlated (*r*=.76), indicating that adolescents who reported higher average levels of school refusal across time also tended to report more severe IGD.

### Multigroup RI-CLPM

**Figure 2.**
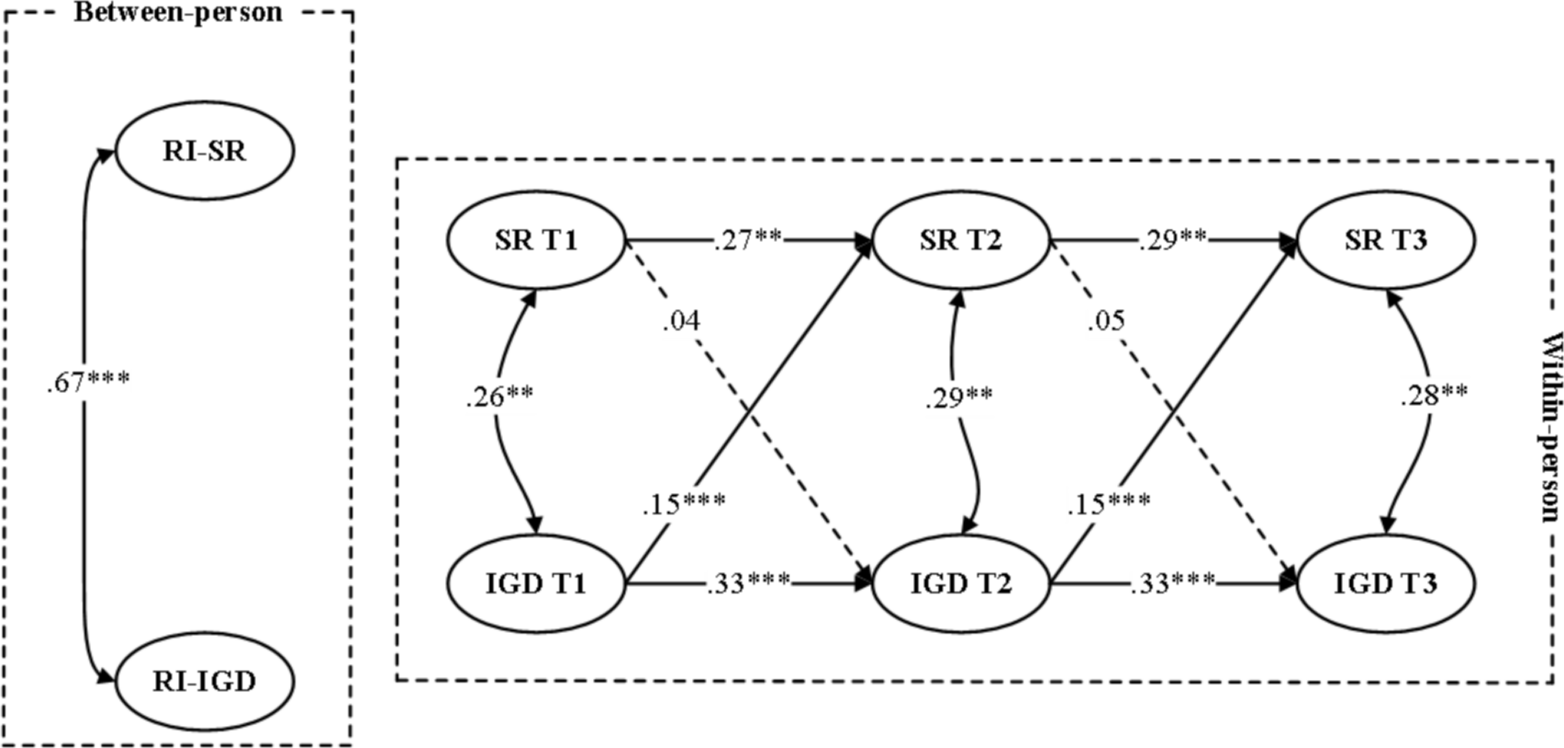
Random intercept (RI) cross-lagged panel model depicting longitudinal associations between school refusal (SR) and internet gaming disorder (IGD) among male adolescent gamers across 3 measurement waves. The model estimates within-person cross-lagged effects while accounting for stable between-person differences. Standardized path coefficients are presented. **P<.01, ***P<.001.

In the first step, an unconstrained model was estimated in which all autoregressive and cross-lagged paths were freely estimated across males and females. The model demonstrated good fit to the data (*χ*^2^_10_=17.86, CFI=0.994, TLI=0.982, RMSEA=0.045, SRMR=0.034). In the second step, a constrained model was tested in which all cross-lagged paths were constrained to be equal across groups. This model showed a less optimal fit (*χ*^2^_14_=48.84, CFI=0.973, TLI=0.943, RMSEA=0.056, SRMR=0.061). A chi-square difference test indicated that the constrained model fit significantly worse than the unconstrained model (Δ*χ*^2^_4_=30.98, *P*<.001), suggesting that the within-person association between school refusal and IGD differed significantly by sex. Figures2  3 present the standardized results.

**Figure 3.**
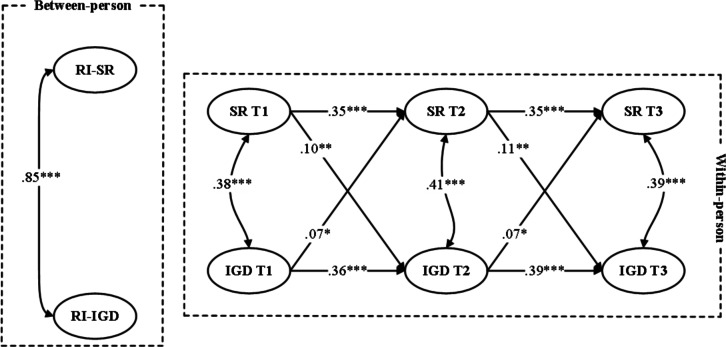
Random intercept (RI) cross-lagged panel model depicting longitudinal associations between school refusal (SR) and internet gaming disorder (IGD) among female adolescent gamers across 3 measurement waves. The model separates within-person temporal effects from between-person stability. Standardized path coefficients are presented. *P<.05, **P<.01, ***P<.001.

Specifically, sex differences were observed in the pathway from school refusal predicting subsequent IGD. A Wald test of parameter constraints indicated a significant difference between males and females (Wald *χ*^2^(1)=10.74, *P*=.001). Among females, school refusal significantly predicted later IGD at both time intervals (T1 → T2: *β*=.10, 95% CI .05-.15; *P*=.006 and T2 → T3: *β*=.11, 95% CI .04-.17; *P*=.004). In contrast, for males, the corresponding cross-lagged paths were not statistically significant. Sex differences were also found in the reverse pathway from IGD predicting school refusal, with a Wald test indicating a significant difference across sexes (Wald test estimate=8.96, *P*=.003). For males, the paths from IGD to later school refusal were significant (T1 → T2: *β*=.15, 95% CI .12-.18; *P*<.001 and T2 → T3: *β*=.15, 95% CI .12-.18; *P*<.001). Among females, IGD significantly predicted school refusal (T1 → T2: *β*=.07, 95% CI .03-.10; *P*=.02 and T2 → T3: *β*=.07, 95% CI .03-.10; *P*=.02), but the effects were weaker.

## Discussion

### Principal Findings

The present study used RI-CLPM to investigate the longitudinal association between school refusal and IGD among Chinese adolescent gamers. By isolating within-person fluctuations from stable between-person differences, this study offers the novel longitudinal evidence that these 2 behaviors co-develop over time in this population. Across 3 waves, school refusal was consistently associated with IGD at the subsequent wave, while IGD was likewise associated with subsequent increases in school refusal. Although the cross-lagged effects were modest in magnitude, their consistency across waves suggests a reliable within-person association. Multigroup RI-CLPM analyses indicated that females showed a significant path from school refusal to later IGD, whereas this path was not significant for males. In the opposite direction, IGD predicted later school refusal for both sexes, but the effects were stronger for males than for females. These findings suggest that school refusal and IGD are associated over time, with the direction and strength of these associations differing by sex.

Using RI-CLPM, the present study revealed significant within-person associations between school refusal and IGD across 3 waves. By separating stable between-person differences, the model captures how fluctuations relative to one’s typical level are associated across time. The results showed that when adolescents reported higher-than-usual levels of school refusal, they also tended to report higher-than-usual levels of IGD at the subsequent wave. Likewise, when adolescents experienced temporary increases in IGD, they tended to show higher levels of school refusal at the next measurement point. Recent methodological work by Orth et al [49] suggests that cross-lagged effects of approximately 0.03, 0.07, and 0.12 can be interpreted as small, medium, and large, respectively, in longitudinal panel models. Within this framework, the association from school refusal to later IGD (*β*=.06) represents a small-to-medium effect, whereas the association from IGD to later school refusal (*β* .12‐.13) reaches the medium-to-large range. Considering that RI-CLPM typically yields conservative estimates, these coefficients suggest that the associations are meaningful at the within-person level rather than trivial.

The within-person association from school refusal to later IGD is consistent with the logic of the escape-avoidance framework and supports H1. Among adolescent gamers, school refusal often reflects attempts to disengage from distressing or overwhelming school-related contexts, such as academic pressure, teacher criticism, peer rejection, or bullying [50]. Avoiding school temporarily removes adolescents from environments that evoke anxiety, shame, or fear of failure and simultaneously creates large blocks of unstructured time with minimal external demands [13]. Online gaming becomes an appealing outlet in such circumstances because it offers immediate feedback, clear goals, rapid progress, and a sense of efficacy that contrasts with the uncertainty and evaluative nature of school settings [51]. When adolescents experience a within-person surge in school refusal, the psychological discomfort underlying avoidance, combined with increased discretionary time, may heighten the likelihood of turning to online games as a form of emotional relief and distraction [52]. Although the effect size for this pathway is small to medium, its repeated appearance across waves suggests a stable association. The finding emerging under the conservative RI-CLPM model further indicates that increases in school-related avoidance tend to be followed by increases in problematic gaming.

The reverse association, that is, from IGD to later school refusal, was stronger in magnitude and aligns with a self-determination perspective on adolescent gaming motivations, supporting H2. Online games often provide highly rewarding compensatory experiences, giving adolescents feelings of competence, relatedness, and autonomy that may be less accessible in the school environment [53]. As IGD intensifies, adolescents may increasingly structure their daily routines, emotional needs, and social relationships around virtual spaces [54]. Activities such as coordinating with guilds or teams, maintaining rankings, or progressing within a game’s achievement system may become central to their identity and self-worth [55]. Under such circumstances, attending school can feel not merely unappealing but actively disruptive. School attendance interrupts gaming activities, reduces contact with gaming peers, and may expose adolescents to criticism about their gaming habits from adults [56]. Consequently, when adolescents exhibit within-person increases in IGD symptoms, school avoidance may function as a strategy to protect the psychological rewards and social belonging embedded in the gaming environment. The medium-to-large effect size suggests that increases in IGD are subsequently followed by higher school refusal.

These reciprocal associations suggest that increases in school refusal and IGD tend to occur together, creating a pattern that may become more stable over time if not addressed. Although each wave-to-wave association is modest in size, the fact that these associations appear consistently across multiple waves suggests that their effects may accumulate. During adolescence, even relatively small but persistent shifts in everyday behavior can, over the course of a school year, influence students’ engagement with school, relationships with peers, and general well-being [57]. Rather than viewing school refusal and IGD as separate concerns, the present findings indicate that they often co-occur and may reflect a broader process through which adolescents manage stress and seek more controllable or rewarding environments. This close connection between the 2 behaviors highlights the value of approaches that consider both behaviors together rather than treating them as unrelated issues.

The *t* test results showed a familiar pattern often reported in previous studies. Across the 3 waves, males had lower mean scores on most dimensions of school refusal (except EAS at T1), whereas they consistently scored higher than females on IGD [34,37]. These mean-level differences suggest that females may be more likely to respond to school-related stress with emotional withdrawal, while males tend to spend more time in gaming. However, mean differences alone cannot tell us whether the 2 behaviors are associated in the same way for males and females over time. For this reason, a multigroup RI-CLPM was performed to examine whether the longitudinal association differed by sex.

The multigroup RI-CLPM results revealed clear sex-specific patterns in the longitudinal associations between school refusal and IGD, as confirmed by Wald tests comparing constrained and unconstrained models. Overall, the findings suggest a more reciprocal pattern among females, whereas for males, the dominant direction runs from IGD to subsequent school refusal, supporting H3. For females, school refusal significantly predicted later IGD at both intervals, whereas this longitudinal path was not significant for males. This indicates that school-related avoidance may play a more central role in the development of problematic gaming among females. In line with the escape-avoidance perspective, females may be more inclined to use gaming as a way of managing school-related distress, such as anxiety about performance, worry about teacher evaluation, or tension in peer relationships [58]. When school experiences become emotionally overwhelming, online games may offer a relatively safe and controllable context, making this coping function more common for females [59]. Importantly, this pattern remained after accounting for stable differences between individuals. This suggests that for females, short-term increases in school-related avoidance are followed by increases in IGD rather than merely reflecting general vulnerability.

In contrast, short-term increases in school refusal among males were not associated with subsequent within-person changes in IGD, despite males reporting higher levels of IGD on average. This pattern suggests that for males, school refusal and gaming are not associated in the same “avoid school then turn to games” sequence that appears for females. Instead, the reverse direction was more evident: for males, IGD significantly predicted later school refusal at both intervals, whereas for females, the same paths were significant but weaker. This pattern implies that among males, periods of higher IGD are more strongly tied to later problems with school participation. One possible interpretation is that males may be more likely to organize their daily routines around gaming. They may stay up late to play, give priority to in-game commitments such as matches or team activities, and gradually reduce the time and energy available for schoolwork and attendance [60]. Over time, these shifts in time use and focus may make school feel less relevant or more burdensome, increasing the likelihood of school refusal when gaming becomes difficult to balance with academic demands [61]. For females, IGD was also associated with later school refusal, but the weaker effect suggests that problematic gaming is less central as a driver of school avoidance than it is for males.

The findings have several implications. First, the reciprocal within-person association between school refusal and IGD suggests that these behaviors should not be treated as independent problems. When adolescents show shifts in either school-related avoidance or gaming involvement, these changes may influence the other domain, which indicates that interventions may be more effective when targeting both areas simultaneously. Second, the sex-specific results further highlight the need for more individualized approaches. For females, providing support to help them manage academic stress, improve classroom experiences, and feel more connected at school may reduce the likelihood of turning to gaming as a coping strategy [62]. For males, paying attention to patterns such as sleep disruption, late-night play, and reduced time for schoolwork may help prevent later school disengagement [63]. More broadly, the study shows that RI-CLPM can help identify how short-term changes in adolescents’ school and gaming experiences relate to each other over time.

### Conclusion

The present study used RI-CLPM to examine how school refusal and IGD develop over time among Chinese adolescent gamers. By separating within-person changes from stable between-person differences, the findings provide evidence of a reinforcing within-person association. The multigroup RI-CLPM further showed that this bidirectional pattern was evident among females, who showed significant paths in both directions. In contrast, males showed a different pattern: IGD predicted later school refusal, but school refusal did not predict later IGD. Given the observational design and the modest effect sizes, these findings should be interpreted cautiously and as indicative of developmental tendencies rather than deterministic processes. These findings provide a clear longitudinal pattern of mutual reinforcement and highlight the importance of considering school disengagement and problematic gaming together rather than as separate issues. The results also show that these processes develop differently for males and females. This suggests important implications for tailored prevention and intervention efforts, as sex-specific differences should be taken into account.

## Supplementary material

10.2196/89619 Multimedia Appendix 1 Tables displaying measurement invariance across sex and fit statistics and model comparisons for random intercept cross-lagged panel models (RI-CLPMs) of school refusal and internet gaming disorder (IGD).

## Abbreviations

ANA: avoidance of negative affectivity
AS: attention-seeking
CFI: comparative fit index
*DSM-5*: *Diagnostic and Statistical Manual of Mental Disorders, Fifth Edition*
EAS: escape from aversive situations
IGD: internet gaming disorder
PTR: pursuit of tangible rewards
RI-CLPM: random intercept cross-lagged panel model
RMSEA: root mean square error of approximation
SRAS-R: School Refusal Assessment Scale-Revised
SRMR: standardized root mean square residual
TLI: Tucker-Lewis Index
