# Supplementary material for: Longitudinal Association Between Internet Gaming Disorder and School Refusal Among Adolescents Using a Random Intercept Cross-Lagged Panel Model: Three-Wave Prospective Cohort Study
Source: JMIR Serious Games. 2026 Mar 4;14:e89619. doi: 10.2196/89619 (PMC12979252; doi:10.2196/89619)
Supplement: Multimedia Appendix 1 [file games-v14-e89619-s001.docx]

Supplementary Table 1 Measurement invariance across sex

| **Variable** | **Model** | **CFI** | **RMSEA** | **SRMR** | **ΔCFI** | **ΔRMSEA** | **ΔSRMR** |
| --- | --- | --- | --- | --- | --- | --- | --- |
| School refusal | Configural Invariance | 0.956 | 0.054 | 0.029 | - | - | - |
|  | Metric Invariance | 0.954 | 0.057 | 0.028 | 0.002 | 0.003 | 0.001 |
|  | Scalar Invariance | 0.957 | 0.059 | 0.032 | 0.003 | 0.002 | 0.004 |
| IGD | Configural Invariance | 0.947 | 0.037 | 0.033 | - | - | - |
|  | Metric Invariance | 0.951 | 0.029 | 0.036 | 0.004 | 0.008 | 0.003 |
|  | Scalar Invariance | 0.952 | 0.027 | 0.033 | 0.001 | 0.002 | 0.003 |

IGD = Internet gaming disorder.

Supplementary Table 2 Fit statistics and model comparisons for RI-CLPMs of school refusal and IGD

| Models | χ^2^ | *df* | RMSEA | CFI | AIC | BIC | Δχ^2^ | Δ*df* | *p* |
| --- | --- | --- | --- | --- | --- | --- | --- | --- | --- |
| M1: Baseline Model (unconstrained model) | 68.89 | 24 | 0.058 | 0.945 | 39632.17 | 38986.65 | - | - | - |
| M2: Model with cross-lagged paths fixed to be time-invariant | 38.74 | 10 | 0.047 | 0.952 | 34524.58 | 35484.63 | 30.15 | 14 | .007 |
| **M3: Model with autoregressive and cross-lagged paths fixed to be time-invariant** | **16.02** | **5** | **0.035** | **0.970** | **30679.36** | **30781.98** | **22.72** | **5** | < **.001** |

Note: Bold indicates the best model.
